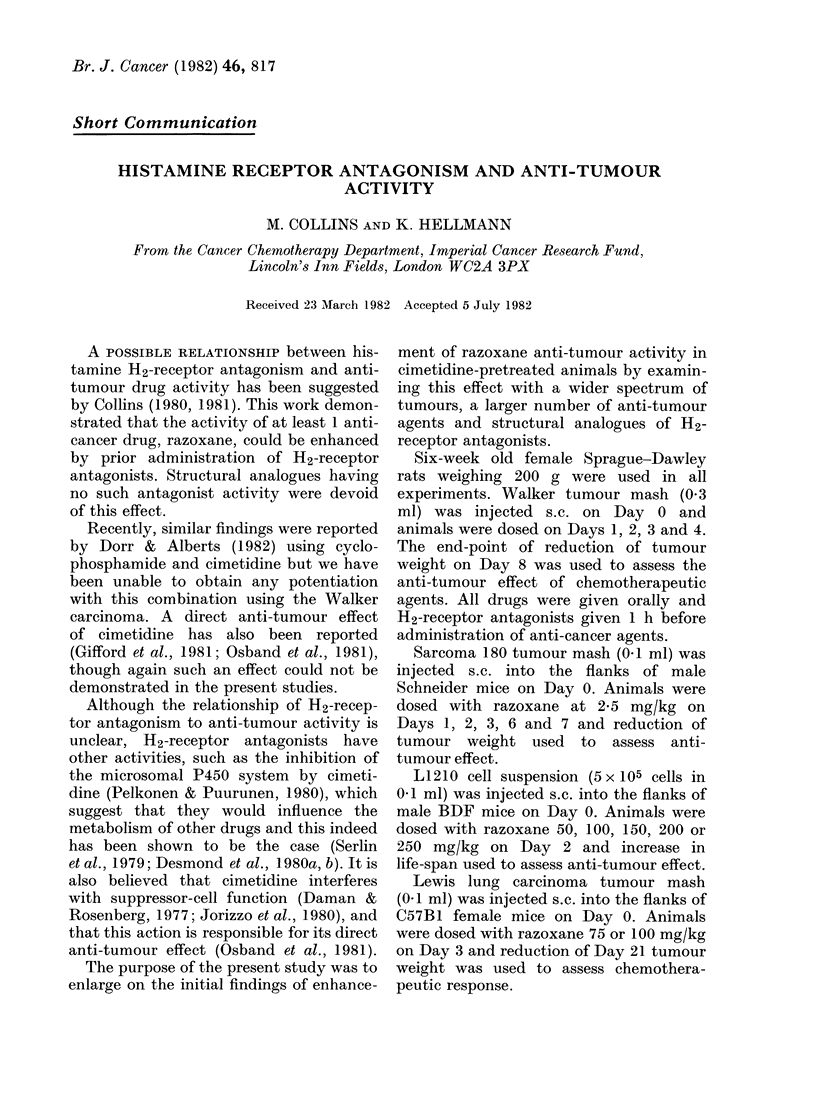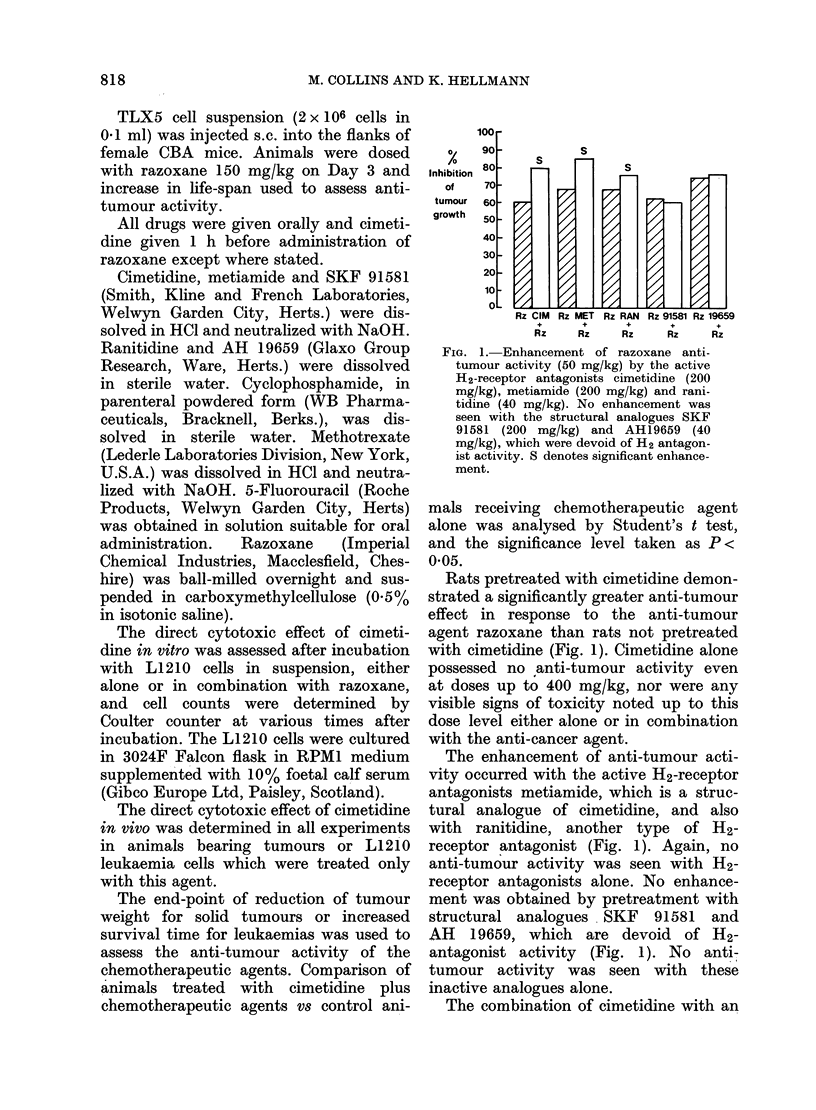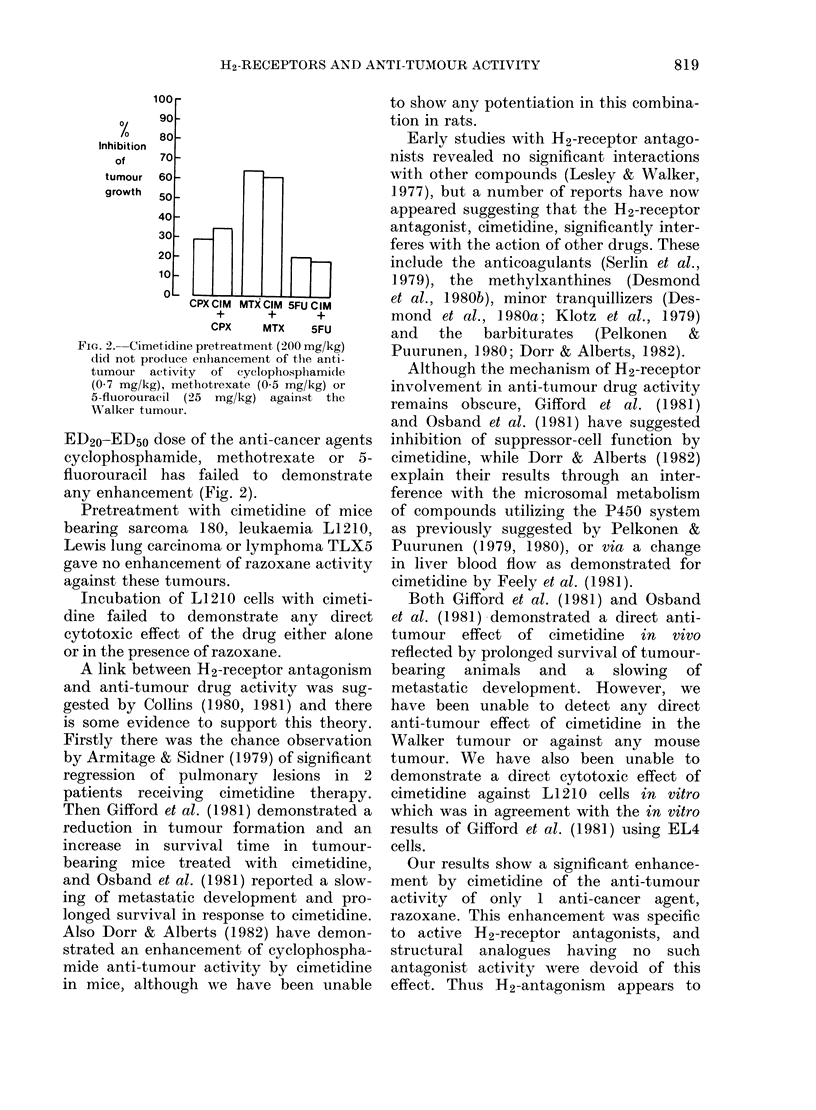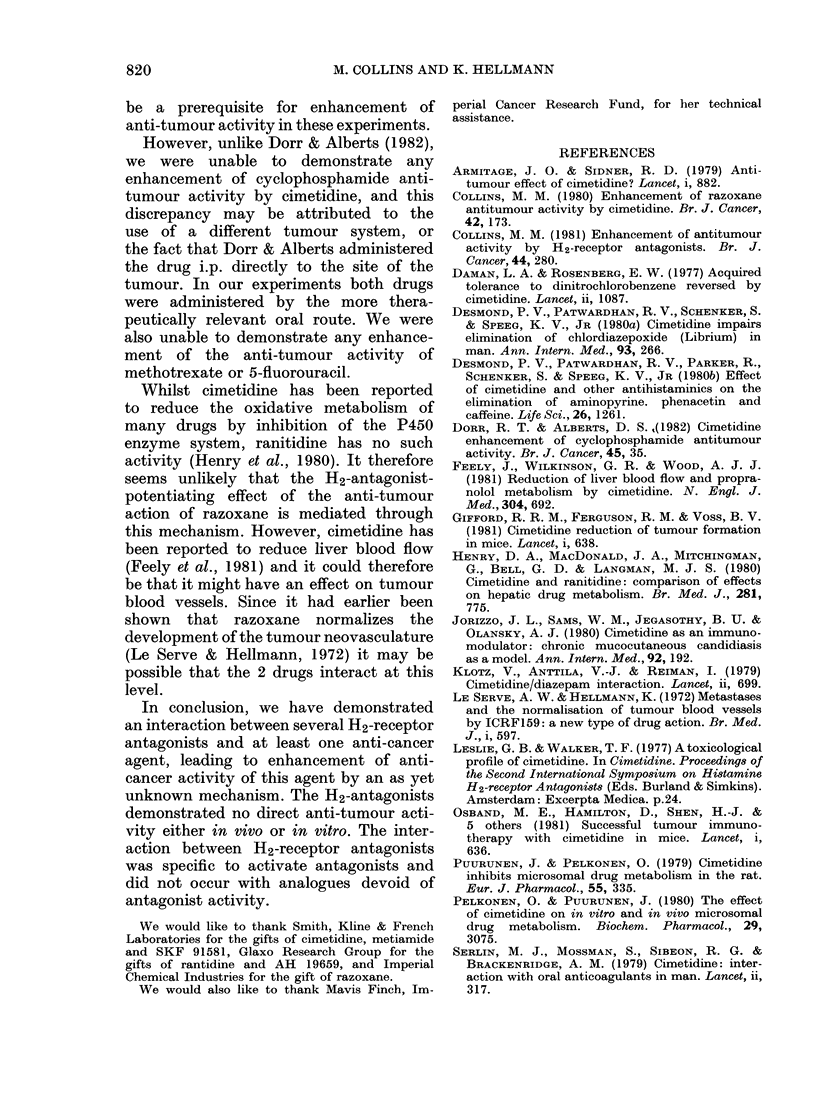# Histamine receptor antagonism and anti-tumour activity.

**DOI:** 10.1038/bjc.1982.276

**Published:** 1982-11

**Authors:** M. Collins, K. Hellmann


*Br. J. Cancer* (1982) **46,** 817

***Short Communication***

### HISTAMINE RECEPTOR ANTAGONISM AND ANTI-TUMOUR ACTIVITY

M. COLLINS AND K. HELLMANN

*From the Cancer Chemotherapy Department, Imperial Cancer Research Fund, Lincoln's Inn Fields, London WC2A 3PX*

Received 23 March 1982 Accepted 5 July 1982

A POSSIBLE RELATIONSHIP between histamine $H_2$-receptor antagonism and anti-tumour drug activity has been suggested by Collins (1980, 1981). This work demonstrated that the activity of at least 1 anti-cancer drug, razoxane, could be enhanced by prior administration of $H_2$-receptor antagonists. Structural analogues having no such antagonist activity were devoid of this effect.

Recently, similar findings were reported by Dorr & Alberts (1982) using cyclophosphamide and cimetidine but we have been unable to obtain any potentiation with this combination using the Walker carcinoma. A direct anti-tumour effect of cimetidine has also been reported (Gifford *et al.*, 1981; Osband *et al.*, 1981), though again such an effect could not be demonstrated in the present studies.

Although the relationship of $H_2$-receptor antagonism to anti-tumour activity is unclear, $H_2$-receptor antagonists have other activities, such as the inhibition of the microsomal P450 system by cimetidine (Pelkonen & Puurunen, 1980), which suggest that they would influence the metabolism of other drugs and this indeed has been shown to be the case (Serlin *et al.*, 1979; Desmond *et al.*, 1980*a*, *b*). It is also believed that cimetidine interferes with suppressor-cell function (Daman & Rosenberg, 1977; Jorizzo *et al.*, 1980), and that this action is responsible for its direct anti-tumour effect (Osband *et al.*, 1981).

The purpose of the present study was to enlarge on the initial findings of enhancement of razoxane anti-tumour activity in cimetidine-pretreated animals by examining this effect with a wider spectrum of tumours, a larger number of anti-tumour agents and structural analogues of $H_2$-receptor antagonists.

Six-week old female Sprague–Dawley rats weighing 200 g were used in all experiments. Walker tumour mash (0·3 ml) was injected s.c. on Day 0 and animals were dosed on Days 1, 2, 3 and 4. The end-point of reduction of tumour weight on Day 8 was used to assess the anti-tumour effect of chemotherapeutic agents. All drugs were given orally and $H_2$-receptor antagonists given 1 h before administration of anti-cancer agents.

Sarcoma 180 tumour mash (0·1 ml) was injected s.c. into the flanks of male Schneider mice on Day 0. Animals were dosed with razoxane at 2·5 mg/kg on Days 1, 2, 3, 6 and 7 and reduction of tumour weight used to assess anti-tumour effect.

L1210 cell suspension ($5 \times 10^5$ cells in 0·1 ml) was injected s.c. into the flanks of male BDF mice on Day 0. Animals were dosed with razoxane 50, 100, 150, 200 or 250 mg/kg on Day 2 and increase in life-span used to assess anti-tumour effect.

Lewis lung carcinoma tumour mash (0·1 ml) was injected s.c. into the flanks of C57Bl female mice on Day 0. Animals were dosed with razoxane 75 or 100 mg/kg on Day 3 and reduction of Day 21 tumour weight was used to assess chemotherapeutic response.

TLX5 cell suspension ($2 \times 10^6$ cells in 0·1 ml) was injected s.c. into the flanks of female CBA mice. Animals were dosed with razoxane 150 mg/kg on Day 3 and increase in life-span used to assess anti-tumour activity.

All drugs were given orally and cimetidine given 1 h before administration of razoxane except where stated.

Cimetidine, metiamide and SKF 91581 (Smith, Kline and French Laboratories, Welwyn Garden City, Herts.) were dissolved in HCl and neutralized with NaOH. Ranitidine and AH 19659 (Glaxo Group Research, Ware, Herts.) were dissolved in sterile water. Cyclophosphamide, in parenteral powdered form (WB Pharmaceuticals, Bracknell, Berks.), was dissolved in sterile water. Methotrexate (Lederle Laboratories Division, New York, U.S.A.) was dissolved in HCl and neutralized with NaOH. 5-Fluorouracil (Roche Products, Welwyn Garden City, Herts) was obtained in solution suitable for oral administration. Razoxane (Imperial Chemical Industries, Macclesfield, Cheshire) was ball-milled overnight and suspended in carboxymethylcellulose (0·5% in isotonic saline).

The direct cytotoxic effect of cimetidine *in vitro* was assessed after incubation with L1210 cells in suspension, either alone or in combination with razoxane, and cell counts were determined by Coulter counter at various times after incubation. The L1210 cells were cultured in 3024F Falcon flask in RPMI medium supplemented with 10% foetal calf serum (Gibco Europe Ltd, Paisley, Scotland).

The direct cytotoxic effect of cimetidine *in vivo* was determined in all experiments in animals bearing tumours or L1210 leukaemia cells which were treated only with this agent.

The end-point of reduction of tumour weight for solid tumours or increased survival time for leukaemias was used to assess the anti-tumour activity of the chemotherapeutic agents. Comparison of animals treated with cimetidine plus chemotherapeutic agents *vs* control animals receiving chemotherapeutic agent alone was analysed by Student's *t* test, and the significance level taken as $P<0.05$.

FIG. 1.—Enhancement of razoxane anti-tumour activity (50 mg/kg) by the active $H_2$-receptor antagonists cimetidine (200 mg/kg), metiamide (200 mg/kg) and ranitidine (40 mg/kg). No enhancement was seen with the structural analogues SKF 91581 (200 mg/kg) and AH19659 (40 mg/kg), which were devoid of $H_2$ antagonist activity. S denotes significant enhancement.

Rats pretreated with cimetidine demonstrated a significantly greater anti-tumour effect in response to the anti-tumour agent razoxane than rats not pretreated with cimetidine (Fig. 1). Cimetidine alone possessed no anti-tumour activity even at doses up to 400 mg/kg, nor were any visible signs of toxicity noted up to this dose level either alone or in combination with the anti-cancer agent.

The enhancement of anti-tumour activity occurred with the active $H_2$-receptor antagonists metiamide, which is a structural analogue of cimetidine, and also with ranitidine, another type of $H_2$-receptor antagonist (Fig. 1). Again, no anti-tumour activity was seen with $H_2$-receptor antagonists alone. No enhancement was obtained by pretreatment with structural analogues SKF 91581 and AH 19659, which are devoid of $H_2$-antagonist activity (Fig. 1). No anti-tumour activity was seen with these inactive analogues alone.

The combination of cimetidine with an

FIG. 2.—Cimetidine pretreatment (200 mg/kg) did not produce enhancement of the anti-tumour activity of cyclophosphamide (0·7 mg/kg), methotrexate (0·5 mg/kg) or 5-fluorouracil (25 mg/kg) against the Walker tumour.

$ED_{20}$–$ED_{50}$ dose of the anti-cancer agents cyclophosphamide, methotrexate or 5-fluorouracil has failed to demonstrate any enhancement (Fig. 2).

Pretreatment with cimetidine of mice bearing sarcoma 180, leukaemia L1210, Lewis lung carcinoma or lymphoma TLX5 gave no enhancement of razoxane activity against these tumours.

Incubation of L1210 cells with cimetidine failed to demonstrate any direct cytotoxic effect of the drug either alone or in the presence of razoxane.

A link between $H_2$-receptor antagonism and anti-tumour drug activity was suggested by Collins (1980, 1981) and there is some evidence to support this theory. Firstly there was the chance observation by Armitage & Sidner (1979) of significant regression of pulmonary lesions in 2 patients receiving cimetidine therapy. Then Gifford *et al.* (1981) demonstrated a reduction in tumour formation and an increase in survival time in tumour-bearing mice treated with cimetidine, and Osband *et al.* (1981) reported a slowing of metastatic development and prolonged survival in response to cimetidine. Also Dorr & Alberts (1982) have demonstrated an enhancement of cyclophosphamide anti-tumour activity by cimetidine in mice, although we have been unable to show any potentiation in this combination in rats.

Early studies with $H_2$-receptor antagonists revealed no significant interactions with other compounds (Lesley & Walker, 1977), but a number of reports have now appeared suggesting that the $H_2$-receptor antagonist, cimetidine, significantly interferes with the action of other drugs. These include the anticoagulants (Serlin *et al.*, 1979), the methylxanthines (Desmond *et al.*, 1980*b*), minor tranquillizers (Desmond *et al.*, 1980*a*; Klotz *et al.*, 1979) and the barbiturates (Pelkonen & Puurunen, 1980; Dorr & Alberts, 1982).

Although the mechanism of $H_2$-receptor involvement in anti-tumour drug activity remains obscure, Gifford *et al.* (1981) and Osband *et al.* (1981) have suggested inhibition of suppressor-cell function by cimetidine, while Dorr & Alberts (1982) explain their results through an interference with the microsomal metabolism of compounds utilizing the P450 system as previously suggested by Pelkonen & Puurunen (1979, 1980), or *via* a change in liver blood flow as demonstrated for cimetidine by Feely *et al.* (1981).

Both Gifford *et al.* (1981) and Osband *et al.* (1981) demonstrated a direct anti-tumour effect of cimetidine *in vivo* reflected by prolonged survival of tumour-bearing animals and a slowing of metastatic development. However, we have been unable to detect any direct anti-tumour effect of cimetidine in the Walker tumour or against any mouse tumour. We have also been unable to demonstrate a direct cytotoxic effect of cimetidine against L1210 cells *in vitro* which was in agreement with the *in vitro* results of Gifford *et al.* (1981) using EL4 cells.

Our results show a significant enhancement by cimetidine of the anti-tumour activity of only 1 anti-cancer agent, razoxane. This enhancement was specific to active $H_2$-receptor antagonists, and structural analogues having no such antagonist activity were devoid of this effect. Thus $H_2$-antagonism appears to be a prerequisite for enhancement of anti-tumour activity in these experiments.

However, unlike Dorr & Alberts (1982), we were unable to demonstrate any enhancement of cyclophosphamide anti-tumour activity by cimetidine, and this discrepancy may be attributed to the use of a different tumour system, or the fact that Dorr & Alberts administered the drug i.p. directly to the site of the tumour. In our experiments both drugs were administered by the more therapeutically relevant oral route. We were also unable to demonstrate any enhancement of the anti-tumour activity of methotrexate or 5-fluorouracil.

Whilst cimetidine has been reported to reduce the oxidative metabolism of many drugs by inhibition of the P450 enzyme system, ranitidine has no such activity (Henry *et al.*, 1980). It therefore seems unlikely that the $H_2$-antagonist-potentiating effect of the anti-tumour action of razoxane is mediated through this mechanism. However, cimetidine has been reported to reduce liver blood flow (Feely *et al.*, 1981) and it could therefore be that it might have an effect on tumour blood vessels. Since it had earlier been shown that razoxane normalizes the development of the tumour neovasculature (Le Serve & Hellmann, 1972) it may be possible that the 2 drugs interact at this level.

In conclusion, we have demonstrated an interaction between several $H_2$-receptor antagonists and at least one anti-cancer agent, leading to enhancement of anti-cancer activity of this agent by an as yet unknown mechanism. The $H_2$-antagonists demonstrated no direct anti-tumour activity either *in vivo* or *in vitro*. The interaction between $H_2$-receptor antagonists was specific to activate antagonists and did not occur with analogues devoid of antagonist activity.

We would like to thank Smith, Kline & French Laboratories for the gifts of cimetidine, metiamide and SKF 91581, Glaxo Research Group for the gifts of rantidine and AH 19659, and Imperial Chemical Industries for the gift of razoxane.

We would also like to thank Mavis Finch, Imperial Cancer Research Fund, for her technical assistance.

### REFERENCES

Armitage, J. O. & Sidner, R. D. (1979) Anti-tumour effect of cimetidine? *Lancet*, i, 882.

Collins, M. M. (1980) Enhancement of razoxane antitumour activity by cimetidine. *Br. J. Cancer*, **42**, 173.

Collins, M. M. (1981) Enhancement of antitumour activity by $H_2$-receptor antagonists. *Br. J. Cancer*, **44**, 280.

Daman, L. A. & Rosenberg, E. W. (1977) Acquired tolerance to dinitrochlorobenzene reversed by cimetidine. *Lancet*, ii, 1087.

Desmond, P. V., Patwardhan, R. V., Schenker, S. & Speeg, K. V., Jr (1980*a*) Cimetidine impairs elimination of chlordiazepoxide (Librium) in man. *Ann. Intern. Med.*, **93**, 266.

Desmond, P. V., Patwardhan, R. V., Parker, R., Schenker, S. & Speeg, K. V., Jr (1980*b*) Effect of cimetidine and other antihistaminics on the elimination of aminopyrine. phenacetin and caffeine. *Life Sci.*, **26**, 1261.

Dorr, R. T. & Alberts, D. S. ,(1982) Cimetidine enhancement of cyclophosphamide antitumour activity. *Br. J. Cancer*, **45**, 35.

Feely, J., Wilkinson, G. R. & Wood, A. J. J. (1981) Reduction of liver blood flow and propranolol metabolism by cimetidine. *N. Engl. J. Med.*, **304**, 692.

Gifford, R. R. M., Ferguson, R. M. & Voss, B. V. (1981) Cimetidine reduction of tumour formation in mice. *Lancet*, i, 638.

Henry, D. A., MacDonald, J. A., Mitchingman, G., Bell, G. D. & Langman, M. J. S. (1980) Cimetidine and ranitidine: comparison of effects on hepatic drug metabolism. *Br. Med. J.*, **281**, 775.

Jorizzo, J. L., Sams, W. M., Jegasothy, B. U. & Olansky, A. J. (1980) Cimetidine as an immunomodulator: chronic mucocutaneous candidiasis as a model. *Ann. Intern. Med.*, **92**, 192.

Klotz, V., Anttila, V.-J. & Reiman, I. (1979) Cimetidine/diazepam interaction. *Lancet*, ii, 699.

Le Serve, A. W. & Hellmann, K. (1972) Metastases and the normalisation of tumour blood vessels by ICRF159: a new type of drug action. *Br. Med. J.*, i, 597.

Leslie, G. B. & Walker, T. F. (1977) A toxicological profile of cimetidine. In *Cimetidine. Proceedings of the Second International Symposium on Histamine $H_2$-receptor Antagonists* (Eds. Burland & Simkins). Amsterdam: Excerpta Medica. p.24.

Osband, M. E., Hamilton, D., Shen, H.-J. & 5 others (1981) Successful tumour immunotherapy with cimetidine in mice. *Lancet*, i, 636.

Puurunen, J. & Pelkonen, O. (1979) Cimetidine inhibits microsomal drug metabolism in the rat. *Eur. J. Pharmacol.*, **55**, 335.

Pelkonen, O. & Puurunen, J. (1980) The effect of cimetidine on *in vitro* and *in vivo* microsomal drug metabolism. *Biochem. Pharmacol.*, **29**, 3075.

Serlin, M. J., Mossman, S., Sibeon, R. G. & Brackenridge, A. M. (1979) Cimetidine: interaction with oral anticoagulants in man. *Lancet*, ii, 317.